# Design and Manufacturing of a Miniature Double-Circular-Arc Line Gear Pump

**DOI:** 10.3390/mi17020222

**Published:** 2026-02-08

**Authors:** Yangzhi Chen, Yimin Yang, Weitao He, Maoxi Zheng, Xiaoping Xiao

**Affiliations:** 1 School of Mechanical and Automotive Engineering, South China University of Technology, Guangzhou 510641, China; 2 Postdoctoral Program, Zhongshan MLTOR Numerical Control Technology Co., Ltd., Zhongshan 528401, China; 3 School of Mechanical and Energy Engineering, Guangdong Ocean University, Yangjiang 529500, China

**Keywords:** miniature gear pump, line gear, double-circular-arc tooth profile, hob design, flow calculation

## Abstract

Traditional involute gear pumps find it difficult to meet the requirements of miniaturization and high performance because of the undercutting, trapped oil, and flow pulsation. To eliminate the phenomenon of trapped oil and reduce flow pulsation in the miniature gear pump, a novel miniature double-circular-arc line gear (MDLG) and its manufacturing method are proposed. Firstly, based on the spatial curve meshing theory, the tooth flank equation of the MDLG is established, and the design method of the MDLG hob is presented. Then, the instantaneous flow rate of the MDLG pump is analyzed by using the swept-area method. Subsequently, a hobbing machining model is built on the VERICUT virtual simulation platform, and machining experiments are conducted on a hobbing machine. Furthermore, the manufactured MDLGs are inspected at a gear measuring center. Finally, an MDLG pump prototype is developed and machined. The measurement results show that the total cumulative pitch deviations of the machined MDLGs are controlled within 32.1 μm, achieving the ISO 8 accuracy grade. The theoretical calculations and experimental results in this article verify the feasibility of the design and processing of MDLG pumps, providing a reference for the development of high-performance miniature gear pumps.

## 1. Introduction

Miniaturization of products and devices is one of the core trends in modern precision engineering [1]. High-performance miniature pumps are playing an increasingly important role in the field of precise fluid transport, with typical applications including inkjet printing, microfluidic chips, drug delivery systems, etc. [2,3]. Gear pumps have great potential in such applications due to their compact structure, high reliability, and stable output. However, further miniaturization and performance enhancement of gear pumps are now facing fundamental theoretical constraints and engineering challenges.

External gear pumps commonly employ involute gears as transmission components; however, traditional involute gears face two inherent limitations. The first limitation is undercutting [4], which increases the difficulty of machining miniature gears, thereby limiting further reduction in the size of gear pumps. The second limitation is the phenomenon of trapped oil, which induces periodic variation in enclosed volume during meshing [5]. This gives rise to pressure surges and noise, both of which severely impair pump performance. This issue becomes more pronounced in high-speed, high-pressure, and miniaturized applications.

Currently, optimizing gear pump relief grooves is the primary method to address trapped oil issues. Nikolov et al. [6] proposed a 2D computational fluid dynamics (CFD) model that incorporates relief grooves for precise simulation of the pump’s design characteristics. Zhang et al. [7] analyzed the effects of relief groove spacing in bidirectional gear pumps on trapped oil pressure, cavitation, and flow pulsation, using CFD simulations with dynamic meshing and experimental validation to identify the optimal spacing. While relief grooves partially alleviate trapped oil, they reduce volumetric efficiency and cannot fully eliminate flow pulsation. These limitations hinder further performance improvements and increase the manufacturing complexity and cost of pump bodies.

To address the root cause of trapped oil, many researchers have focused on refining tooth profiles. Chen et al. [8] proposed that using an arc tooth profile with a transverse contact ratio not exceeding 1 can theoretically resolve the trapped oil issues in involute gears. Zhou et al. [9] developed a gear pair with an “arc-involute-arc” tooth profile featuring a transverse contact ratio of 0.5 and investigated the performance of this gear pump under high-speed, high-pressure conditions. Their results showed that the circular-arc gear pump exhibited no trapped oil and minimal fluctuations in outlet pressure. Zhao et al. [10,11] proposed a numerical method to analyze the kinematic flow ripple of the “arc-involute-arc” gear pump, showing that this design can reduce or even eliminate kinematic flow ripple. Li et al. [12] employed CFD methods to analyze flow pulsations in a circular-arc gear pump. Results indicate that when the module and number of teeth are appropriately designed, flow pulsations and noise are minimal. Huang et al. [13,14] introduced a microsegment gear pump incorporating circular-arc gear design principles and validated through CFD analysis that this design reduces pressure pulsations under high-speed, high-pressure operating conditions. However, these gear pumps were designed based on the theory of spatial conjugate surface meshing. During meshing, the instantaneous meshing area changes periodically as the gears rotate, potentially causing outlet pressure pulsation [15].

**Figure 1:**
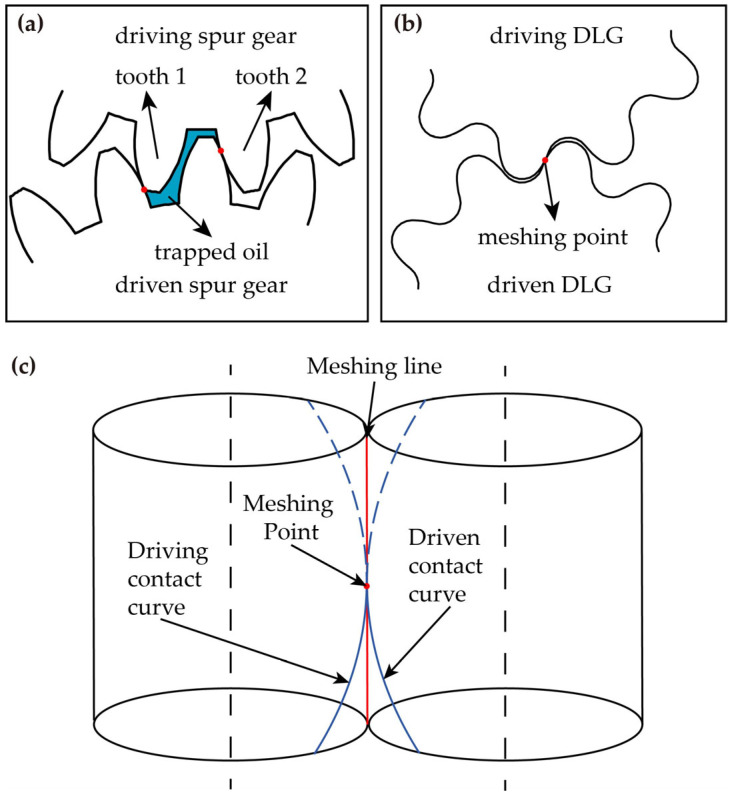
Schematic diagram of the non-trapped oil principle: (**a**) Contact state of spur gear; (**b**) Contact state of DLG; (**c**) Schematic diagram of continuous point meshing of DLG pair.

Line gears (LGs) are a novel gear type based on spatial conjugate curve meshing theory [16]. A parallel-axis LG features a small number of teeth, point contact, pure rolling motion [17,18,19], and zero transverse contact ratio. These characteristics make LGs promising for miniaturized and lightweight machinery applications. Chen et al. [20] designed pure rolling gear pumps with different tooth profiles. CFD analysis results indicate that the pure rolling gear pump with a parabolic tooth profile exhibits lower pressure pulsation. Chen et al. [21] proposed a tooth profile design method for double-circular-arc line gear (DLG) pumps based on LG design principles and completed machining trials using form milling. Performance comparison tests between the DLG pump prototype and a commercial involute gear pump prototype showed that the DLG pump exhibited superior volumetric efficiency and reduced outlet pressure pulsation under identical test conditions. As shown in Figure 1, compared to involute gears, the DLG’s continuous-point contact meshing and zero-transverse contact ratio prevent the formation of enclosed volumes during engagement, while the instantaneous meshing area remains virtually constant. This meshing mechanism theoretically eliminates the trapped oil issues and reduces pressure pulsation, offering significant potential for engineering applications. However, applying LGs to mass-produced miniature pumps remains challenging due to manufacturing constraints. Previous studies have employed 3D printing and computerized numerical control milling for LG manufacturing [22,23,24], but these methods are inefficient and costly, making them unsuitable for mass production of miniature gears.

For miniature gear manufacturing, alternative materials and processes have been explored. Rodionov et al. [25] machined gear pairs with a 0.8 mm module and 15 teeth for miniature gear pumps using PEEK and PPS, demonstrating the feasibility of plastic materials in miniature pump gear applications. Gietzelt et al. [26] fabricated gears with a tip diameter of less than 1 mm for miniature pumps using micro-powder injection molding. However, this method is associated with high manufacturing costs, relatively low precision, and the need for complex post-processing techniques. Gear hobbing, based on the generating method principle, achieves accuracy grades of ISO 7 to ISO 8 by adhering to strict geometric constraints during the hob’s trajectory and relative motion to the gear blank. Moreover, the continuous cutting process eliminates idle strokes, allowing the entire tooth width to be completed in a single pass, making it well-suited for mass production.

The core of gear hobbing lies in the design of high-precision hob cutters. Currently, non-involute gear hob cutters are primarily designed based on the common rack method [27,28] simplifying the complex spatial meshing between the hob and gear into two planar meshing problems. However, these methods lack theoretical rigor and are only applicable when the hob’s lead angle is small, and the required tooth profile accuracy is relatively low. Therefore, developing a theoretically rigorous and precise hob design method directly based on the spatial conjugate relationship between the gear and hob is a critical technical bottleneck for enabling the high-performance, miniaturized application of DLG pumps.

In summary, a miniature double-circular-arc line gear (MDLG) suitable for miniature, low-noise hydraulic applications is introduced in Section 2. The hob design method for the MDLG, based on spatial meshing theory, is presented. In Section 3, the instantaneous flow model for the MDLG pump is derived using the swept-area method, and the calculation results are verified through simulations in SolidWorks 2020. Section 4 tests the accuracy of the designed hob using Vericut 9.2, hobbing experiments, and gear precision testing instruments. Section 5 details the prototype design of the MDLG pump. Finally, Section 6 provides a comprehensive summary of the entire research.

## 2. Design and Hobbing Model for MDLG

### 2.1. Design Methodology for MDLG

LGs are based on the principle of spatial conjugate curve meshing. During transmission, a pair of spatial conjugate curves on the driving and driven gears maintains point contact at all times, with the continuous trajectory formed by the contact points constituting the contact curve. Traditional LG designs typically incorporate a certain amount of tip clearance. In contrast, the tooth tip and root arc segment of the MDLG are designed as a pair of arcs with equal radius and centers located on the pitch circle. This design eliminates tip clearance, creating an effective seal during meshing, which meets the sealing requirements when conveying fluid media.

**Figure 2:**
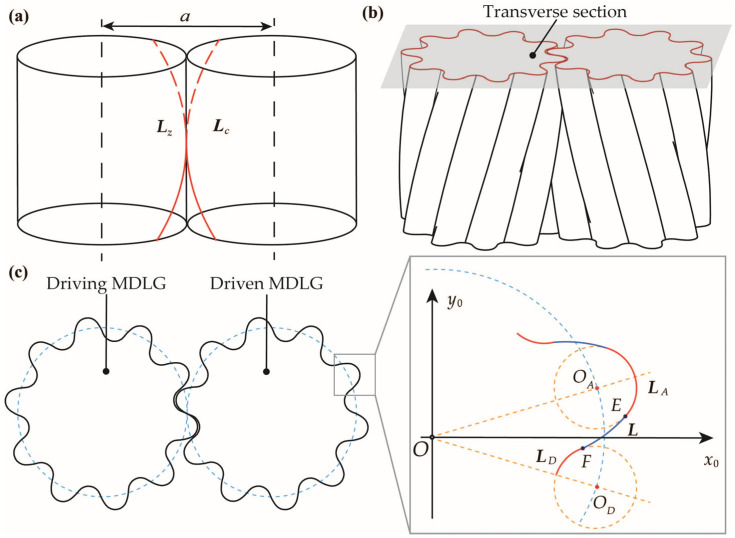
The schematic diagram of the MDLG: (**a**) A pair of meshing line teeth; (**b**) Transverse section of the MDLG; (**c**) Transverse tooth profile of the MDLG.

As shown in Figure 2, the transverse tooth profile of the MDLG is formed by the tooth tip arc (***L***_A_), the tooth root arc (***L***_D_), and the transverse profile of the working tooth flank (***L***). The center of ***L***_A_ is point *O_A_*, and the center of ***L***_D_ is point *O_D_*; both *O_A_* and *O_D_* are located on the pitch circle. Profile ***L*** intersects with ***L***_A_ at point *E* and with ***L***_D_ at point *F*. These three tooth profiles are connected at points *E* and *F* with positional continuity and tangential continuity.

Based on the forming principle and design method of LG, the equations for the driving contact curves (***L****_z_*) and driven contact curves (***L****_c_*) of the MDLG are derived as Equations (1) and (2).

(1) $$L_{z}=\left\{\begin{array}{c} m\cos t\\ m\sin t\\ n\pi +nt \end{array}\right.$$

(2) $$L_{c}=\left\{\begin{array}{c} \left(m-a\right)\cos t\\ \left(a-m\right)\sin t\\ nt \end{array}\right.$$

where *m* and *n* are the radius and pitch of the helix, respectively, and a represents the center distance.

The working tooth flank of the MDLG is generated as follows: select an arc as the generatrix within the normal plane of an arbitrary point on the contact curve ***L****_i_*, then sweep it along the contact curve to form the working tooth flank. The detailed design process can be referenced in [18]. Projecting the working tooth flank onto the transverse section yields the transverse tooth profile equation as Equation (3).

(3) $$L=\left\{\begin{array}{c} {}[m-\rho (\sin\varphi +\cos u)]\cos t+\frac{n\rho (-\cos\varphi +\sin u)}{\sqrt{m^{2}+n^{2}}}\sin t\\ {}[m-\rho (\sin\varphi +\cos u)]\sin t+\frac{n\rho (-\cos\varphi +\sin u)}{\sqrt{m^{2}+n^{2}}}\cos t \end{array}\right.$$

where *ρ* is the radius of the normal arc, *φ* is the normal tooth profile deflection angle, and *u* is the parameter indicating the scope of the normal arc, $t=\frac{m\rho (\cos\varphi -\sin u)}{n\sqrt{m^{2}+n^{2}}}$.

***L****_A_* and ***L****_D_* are designed as equal-radius arcs centered on the pitch circle. Accordingly, the transverse profile equations of the tooth tip arc and the tooth root arc are given by Equations (4) and (5).

(4) $$L_{A}=\left\{\begin{array}{c} m\cos\frac{\pi }{2N_{2}}+r\cos u_{a}\\ m\sin\frac{\pi }{2N_{2}}+r\sin u_{a} \end{array}\right.$$

(5) $$L_{D}=\left(\begin{array}{c} m\cos\frac{\pi }{2N_{2}}+r\cos u_{d}\\ -m\sin\frac{\pi }{2N_{2}}+r\sin u_{d} \end{array}\right)$$

where *r* is the radius of the node center arc, *u_a_* is the central angle of the tooth tip arc, *u_d_* is the central angle of the tooth root arc, and *N*_2_ is the number of teeth for the gear.

Since LG is a point-contact gear; only one working tooth surface point on the pitch circle participates in meshing at any given moment. Therefore, the curvature discontinuities at points *E* and *F* do not affect the normal meshing of MDLG. However, to facilitate manufacturing and avoid interference, positional and tangential continuity conditions must be satisfied at points *E* and *F*, as shown in Equation (6). Specifically, any arc curve must share a common intersection point with the transverse profile curve of the working tooth flank, and the first derivatives at the intersection point must be equal. Based on the continuity condition, the tooth profile parameters, including *φ* and *r*, can be determined.

(6) $$\begin{array}{l} \left\{\begin{array}{c} L_{A}(E)=L(E)\\ L_{D}(F)=L(F) \end{array}\right.\\ \left\{\begin{array}{c} \frac{dL_{A}(E)}{d\theta_{a}}=\frac{dL(E)}{d\theta }\\ \frac{dL_{D}(F)}{d\theta_{d}}=\frac{dL(F)}{d\theta } \end{array}\right. \end{array}$$

By performing a cylindrical helical motion of constant pitch along the contact curve using the transverse profiles as the generatrix, the gear tooth flank of an MDLG can be expressed by Equation (7).

(7) $$\Sigma_{2}=\left\{\begin{array}{l} X_{2}=x_{0}\cos\theta -y_{0}\sin\theta \\ Y_{2}=x_{0}\sin\theta +y_{0}\cos\theta \\ Z_{2}=n\theta \end{array}\right.$$

where *x*_0_ and *y*_0_ represent the transverse profile expressions of the MDLG, *θ* is the parameter of the tooth flank.

For the specific operating conditions and spatial constraints of miniature gear pumps, the basic design parameters for MDLG are set, as shown in Table 1, where ***ζ*** is the contact ratio.

According to the design principles for LG, once the basic parameters are determined, the helix angle (*β*) and tooth width (*B*) can be calculated using Equations (8) and (9)

(8) $$\begin{array}{c} \beta =\arctan(m/n) \end{array}$$

(9) $$B=2\pi n\zeta /N_{2}$$

**Figure 3:**
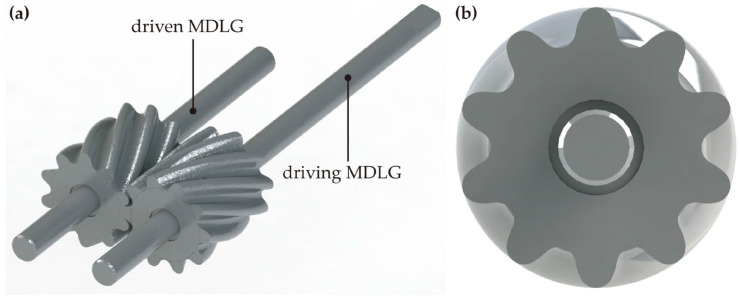
3D models of MDLGs: (**a**) Driving and driven MDLGs; (**b**) Transverse section of MDLG.

Substituting design parameters into Equations (8) and (9) yields *β* = 24.664° and *B* = 12.26 mm. Solving Equation (3) simultaneously with Equation (6) yields *φ* = 23.09° and *r* = 0.725 mm. Substituting these parameters into Equations (3)–(5) produces the transverse profile for a pair of MDLGs. Using this profile as the contour, the gear axis as the path, and the contact curve as the guide curve, a 3D solid model of a single tooth was created in SolidWorks using the “Sweep” function. Finally, the complete driving and driven MDLG models were generated by applying the “Circular Pattern” function to replicate the single tooth, as shown in Figure 3.

### 2.2. Derivation of the Hob Cutter Profile Based on Space Meshing Principle

The design of MDLG hobs can be divided into the following steps:

1. Establish the spatial meshing coordinate system: based on the positional relationship between the MDLG and the hob, establish the spatial meshing coordinate system for the MDLG and the hob, and derive the coordinate transformation matrix from the MDLG to the hob frame.
2. Formulate and solve the spatial meshing equation: based on the kinematic relationship between the MDLG and the hob, derive the relative motion velocity vector and the tooth flank normal vector, then establish and solve the meshing equation accordingly.
3. Determine the axial profile of the hob: transform the contact point parameters obtained from the meshing equation into the hob tooth flank equations via coordinate transformation, define the axial profile based on these equations, and finalize the hob design in accordance with standard hob design methodologies.

#### 2.2.1. Establish the Spatial Meshing Coordinate System

**Figure 4:**
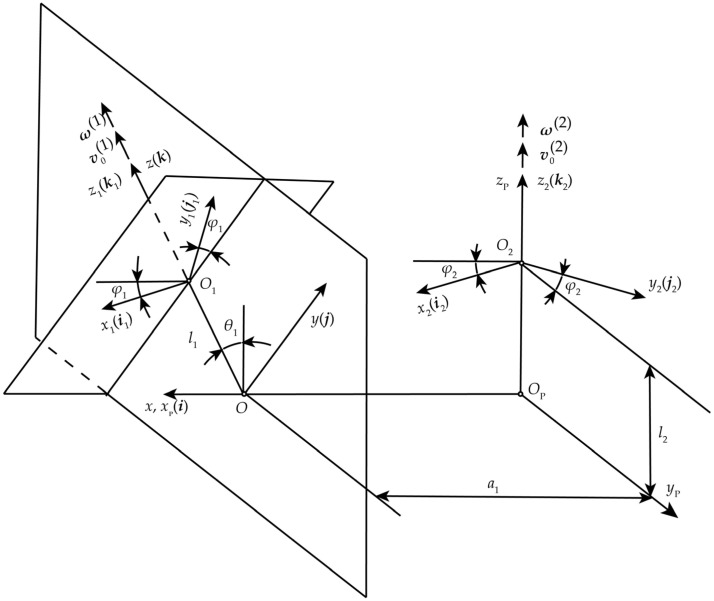
Spatial meshing coordinate system for the MDLG and the hob.

Based on the positional relationship between the MDLG and the hob, the spatial meshing coordinate system for both is established, as shown in Figure 4. The coordinate systems *O-xyz* and *O*_P_-*x*_P_*y*_P_*z*_P_ are two fixed spatial coordinate systems. The *z*-axis coincides with the rotational axis of the hob, while the *z*_P_-axis coincides with the rotational axis of the MDLG. The angle between these two axes is defined as *θ*_1_, and the distance a_1_ between them represents the center distance between the gear and the hob. The *x*-axis is collinear with the *x*_P_-axis. Coordinate system *O*_1_-*x*_1_*y*_1_*z*_1_ is fixed to the hob, and coordinate system *O*_2_-*x*_2_*y*_2_*z*_2_ is fixed to the MDLG. At the initial position, both coincide with the coordinate systems *O-xyz* and *O*_P_-*x*_P_*y*_P_*z*_P_, respectively. The hob rotates around the *z*-axis at a constant angular velocity (***ω***^(1)^) and moves uniformly along the *z*-axis at a constant velocity (***υ***_0_^(1)^). Similarly, the MDLG rotates around the *z*_P_-axis at a constant angular velocity (***ω***^(2)^) and moves uniformly along the *z*_P_-axis at a constant velocity (***υ***_0_^(1)^). After a period of time from the initial position, the coordinate systems *O*_1_-*x*_1_*y*_1_*z*_1_ and *O*_2_-*x*_2_*y*_2_*z*_2_ are displaced to the positions shown in Figure 4, with the distances *OO*_1_ = *l*_1_ and *O*_P_*O*_2_ = *l*_2_. The hob has rotated an angle *φ*_1_ around the *z*-axis, while the MDLG has rotated an angle *φ*_2_ around the *z*_P_-axis.

Transformation matrixes ***M***_10_ from *O-xyz* to *O*_1_-*x*_1_*y*_1_*z*_1_, ***M***_02_ from *O*_2_-*x*_2_*y*_2_*z*_2_ to *O-xyz*, and ***M***_12_ from *O*_2_-*x*_2_*y*_2_*z*_2_ to *O*_1_-*x*_1_*y*_1_*z*_1_ are given by Equations (10)–(12), respectively.

(10) $$M_{10}=\left[\begin{array}{cccc} \cos\varphi_{1} & \sin\varphi_{1} & 0 & 0\\ -\sin\varphi_{1} & \cos\varphi_{1} & 0 & 0\\ 0 & 0 & 1 & -l_{1}\\ 0 & 0 & 0 & 1 \end{array}\right]$$

(11) $$M_{02}=\left[\begin{array}{cccc} \cos\varphi_{2} & -\sin\varphi_{2} & 0 & -a_{1}\\ \sin\varphi_{2}\cos\theta_{1} & \cos\varphi_{2}\cos\theta_{1} & \sin\theta_{1} & l_{2}\sin\theta_{1}\\ -\sin\varphi_{2}\sin\theta_{1} & -\cos\varphi_{2}\sin\theta_{1} & \cos\theta_{1} & l_{2}\cos\theta \\ 0 & 0 & 0 & 1 \end{array}\right]$$

(12) $$\begin{array}{l} M_{12}=M_{10}M_{02}\\ =\left[\begin{array}{cccc} \cos\varphi_{1}\cos\varphi_{2}+\sin\varphi_{1}\sin\varphi_{2}\cos\theta_{1} & -\cos\varphi_{1}\sin\varphi_{2}+\sin\varphi_{1}\cos\varphi_{2}\cos\theta_{1} & -\sin\varphi_{1}\sin\theta_{1} & a_{1}\cos\varphi_{1}-l_{2}\sin\varphi_{1}\sin\theta_{1}\\ -\sin\varphi_{1}\cos\varphi_{2}+\cos\varphi_{1}\sin\varphi_{2}\cos\theta_{1} & \sin\varphi_{1}\sin\varphi_{2}+\cos\varphi_{1}\cos\varphi_{2}\cos\theta_{1} & -\cos\varphi_{1}\sin\theta_{1} & -a_{1}\cos\varphi_{1}-l_{2}\cos\varphi_{1}\sin\theta_{1}\\ -\sin\varphi_{2}\sin\theta_{1} & -\cos\varphi_{2}\sin\theta_{1} & \cos\theta_{1} & l_{2}\cos\theta_{1}-l_{1}\\ 0 & 0 & 0 & 1 \end{array}\right] \end{array}$$

#### 2.2.2. Formulate and Solve the Spatial Meshing Equation

When the MDLG meshes with the hob, the helix surface of the hob and the tooth flank of the gear are tangent to each other at every instant, sharing the same tangent and normal at the point of contact. The meshing equation between the helix surface of the hob and the tooth flank of the MDLG is expressed as Equation (13).

(13) $$v^{\left(12\right)}\cdot n^{\left(2\right)}=0$$

where ***v***^(12)^ is the relative motion velocity between the MDLG and the hob, and ***n***^(2)^ is the normal vector of the tooth flank of the MDLG.

The relative motion velocity ***v***^(12)^ can be expressed in parametric form in the coordinate system *O*_2_-*x*_2_*y*_2_*z*_2_, as given by Equation (14).

(14) $$v^{\left(12\right)}=\left\{\begin{array}{l} v_{x}^{\left(12\right)}=(-\omega_{1}\cos\theta_{1}+\omega_{2})(X_{2}\sin\varphi_{2}+Y_{2}\cos\varphi_{2})-\omega_{1}(Z_{2}+l_{2})\sin\theta_{1}\\ v_{y}^{\left(12\right)}=(-\omega_{2}\cos\theta_{1}+\omega_{1})(X_{2}\cos\varphi_{2}-Y_{2}\sin\varphi_{2})-\omega_{1}a_{1}-v_{02}\sin\theta_{1}\\ v_{z}^{\left(12\right)}=\omega_{2}(X_{2}\cos\varphi_{2}-Y_{2}\sin\varphi_{2})\sin\theta_{1}-v_{01}-v_{02}\cos\theta_{1} \end{array}\right.$$

where *ω*_1_ is the magnitude of the angular velocity of the hob, *ω*_2_ is the magnitude of the angular velocity of the MDLG, *υ*_01_ is the magnitude of the axial translational velocity of the hob, and *υ*_02_ is the magnitude of the axial translational velocity of the MDLG.

The normal vector ***n***^(2)^ at any point on the tooth flank of MDLG is given by Equation (15).

(15) $$n_{2}^{\left(2\right)}=\left\{\begin{array}{c} n_{x2}^{\left(2\right)}=\left|\begin{array}{cc} \frac{\partial Y_{2}}{\partial u} & \frac{\partial Z_{2}}{\partial u}\\ \frac{\partial Y_{2}}{\partial \theta } & \frac{\partial Z_{2}}{\partial \theta } \end{array}\right|\\ n_{y2}^{\left(2\right)}=\left|\begin{array}{cc} \frac{\partial Z_{2}}{\partial u} & \frac{\partial X_{2}}{\partial u}\\ \frac{\partial Z_{2}}{\partial \theta } & \frac{\partial X_{2}}{\partial \theta } \end{array}\right|\\ n_{z2}^{\left(2\right)}=\left|\begin{array}{cc} \frac{\partial X_{2}}{\partial u} & \frac{\partial Y_{2}}{\partial u}\\ \frac{\partial X_{2}}{\partial \theta } & \frac{\partial Y_{2}}{\partial \theta } \end{array}\right| \end{array}\right.$$

where $n_{x2}^{\left(2\right)}$, $n_{y2}^{\left(2\right)}$, $n_{z2}^{\left(2\right)}$ are the components of the normal vector ***n***^(2)^ along the *x*_2_, *y*_2_, and *z*_2_ coordinate axes, respectively.

Then, three components of ***n***^(2)^ in the fixed coordinate system *O-xyz* can be expressed in terms of the parameters of the coordinate system *O*_2_-*x*_2_*y*_2_*z*_2_, as shown in Equation (16).

(16) $$n^{\left(2\right)}=\left\{\begin{array}{l} n_{x}^{\left(2\right)}=n_{x2}^{\left(2\right)}\cos\varphi_{2}-n_{y2}^{\left(2\right)}\sin\varphi_{2}\\ n_{y}^{\left(2\right)}=(n_{x2}^{\left(2\right)}\sin\varphi_{2}+n_{y2}^{\left(2\right)}\cos\varphi_{2})\cos\theta_{1}+n_{x2}^{\left(2\right)}\sin\theta_{1}\\ n_{z}^{\left(2\right)}=-(n_{x2}^{\left(2\right)}\sin\varphi_{2}+n_{y2}^{\left(2\right)}\cos\varphi_{2})\sin\theta_{1}+n_{x2}^{\left(2\right)}\cos\theta_{1} \end{array}\right.$$

**Figure 5:**
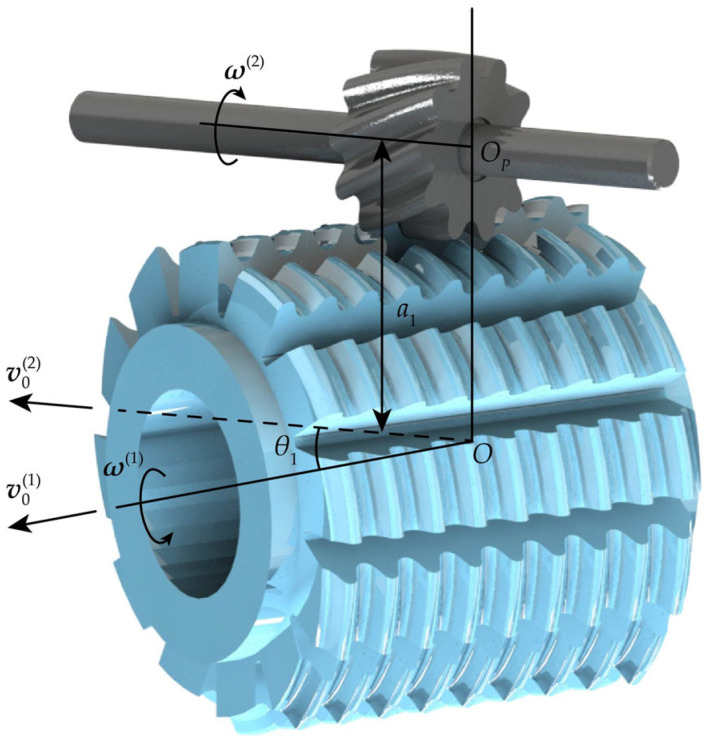
Hobbing motion diagram.

As illustrated in Figure 5, during the hobbing process, the meshing motion parameters include the translational velocity ***v***_01_ along the hob axis, the angular velocity *ω*_1_ of the hob, the angular velocity *ω*_2_ of the MDLG, and the axial movement velocity ***v***_02_ of the MDLG. Among these, the translational motion along the hob axis ***v***_01_ is a theoretical kinematic component required to describe the relative screw motion between the hob and the gear, rather than an actual feed motion. Additionally, the movement of the MDLG along its own helix ***v***_02_ is provided by the machine tool’s differential gear train to ensure the correct lead of the gear and is independent of the instantaneous meshing condition at the contact point. Therefore, ***v***_02_ can be omitted in the meshing equation. Consequently, the motion relationship between the MDLG and the hob can be expressed by Equation (17).

(17) $$\left\{\begin{array}{l} \omega_{1}=\omega_{0}+\frac{v_{01}}{n_{1}}\\ \omega_{2}=i_{21}\omega_{0} \end{array}\right.$$

where *ω*_0_ is the rotational speed of the hob along its own helix, *i*_21_ is the transmission ratio between the MDLG and the hob, and *n*_1_ is the helix parameter of the hob.

Furthermore, substituting the movement relationship into Equation (13) yields simplified expressions as Equation (18).

(18) $$\left\{\begin{array}{c} \begin{array}{l} -i_{21}n_{y2}^{\left(2\right)}X_{2}+i_{21}n_{x2}^{\left(2\right)}Y_{2}-\sin\theta_{1}(a_{1}n_{z2}^{\left(2\right)}-n_{z2}^{\left(2\right)}X_{2}\cos\varphi_{2}+n_{z2}^{\left(2\right)}Y_{2}\sin\varphi_{2}-n_{y2}^{\left(2\right)}Z_{2}\sin\varphi_{2})\\ +\cos\theta_{1}(n_{y2}^{\left(2\right)}X_{2}-n_{x2}^{\left(2\right)}Y_{2}-a_{1}(n_{y2}^{\left(2\right)}\cos\varphi_{2}+n_{x2}^{\left(2\right)}\sin\varphi_{2}))=0 \end{array}\\ \begin{array}{l} -\sin\theta_{1}(a_{1}n_{z2}^{\left(2\right)}+(n_{1}n_{y2}^{\left(2\right)}-n_{z2}^{\left(2\right)}X_{2}+n_{x2}^{\left(2\right)}Z_{2})\cos\varphi_{2}+(n_{1}n_{x2}^{\left(2\right)}+n_{z2}^{\left(2\right)}Y_{2}-n_{y2}^{\left(2\right)}Z_{2})\sin\varphi_{2})\\ +\cos\theta_{1}(n_{y2}^{\left(2\right)}X_{2}-n_{x2}^{\left(2\right)}Y_{2}-a_{1}(n_{y2}^{\left(2\right)}\cos\varphi_{2}+n_{x2}^{\left(2\right)}\sin\varphi_{2}))=0 \end{array} \end{array}\right.$$

After determining the contact point coordinates (*u* and *θ*) at specific values of *φ*_2_ and *l*_1_ from the meshing equation, the meshing surface equation and tooth flank equation can be derived through coordinate transformation.

#### 2.2.3. Determine the Axial Profile of the Hob

The equation for the helix surface of the hob can be derived from the tooth flank equation of the MDLG via the coordinate transformation matrix ***M***_12_, as shown in Equation (19).

(19) $$\Sigma_{1}=\left\{\begin{array}{l} X_{1}=-\cos\varphi_{1}\left(a_{1}-X_{2}\cos\varphi_{2}+Y_{2}\sin\varphi_{2}\right)+\sin\varphi_{1}\left(Z_{2}\sin\theta_{1}+\cos\theta_{1}\left(Y_{2}\cos\varphi_{2}+X_{2}\sin\varphi_{2}\right)\right)\\ Y_{1}=Z_{2}\cos\varphi_{1}\sin\theta_{1}+\cos\theta_{1}\cos\varphi_{1}\left(X_{2}\sin\varphi_{2}+Y_{2}\cos\varphi_{2}\right)+\sin\varphi_{1}\left(a_{1}-X_{2}\cos\varphi_{2}+Y_{2}\sin\varphi_{2}\right)\\ Z_{1}=-l_{1}+Z_{2}\cos\theta_{1}-Y_{2}\cos\varphi_{2}\sin\theta_{1}-X_{2}\sin\theta_{1}\sin\varphi_{2} \end{array}\right.$$

By setting *Y*_1_ to 0, the relationship between *l*_1_ and *φ*_2_ can be established. Substituting this into the hob’s helix equation allows the axial profile of the hob to be determined.

#### 2.2.4. Modeling of the Hob Cutter

The primary design parameters of the hob are listed in Table 2.

**Figure 6:**
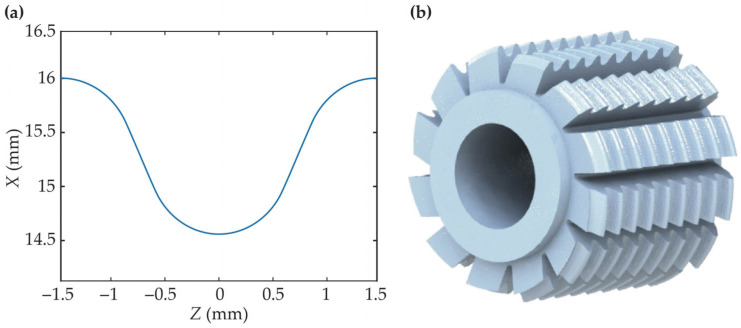
MDLG hob: (**a**) axial profile of the hob; (**b**) 3D model of the hob.

**Figure 7:**
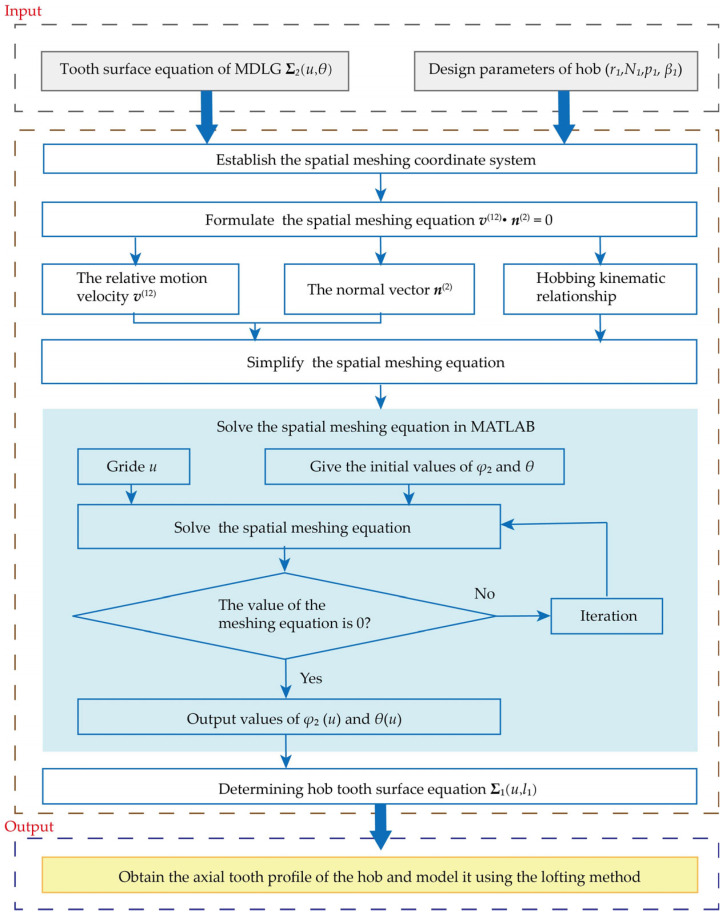
Flowchart of solving algorithm for MDLG hob.

Substituting the parameters from Table 2 into Equation (19) yields the axial profile of the hob. This profile is plotted using Matlab 2020, as shown in Figure 6a. After obtaining the data points of the hob’s axial profile, a macro program in SolidWorks 2020 is invoked to generate the corresponding helical curves based on these points. A loft operation is then performed on these helical curves to form the helical surface of the hob. Subsequently, through surface trimming and surface knitting, the solid body of the hob is obtained. Following the general methodology for hob design, slotting and backing-off operations are applied to complete the 3D model of the hob, as illustrated in Figure 6b. In summary, the flowchart of the solving algorithm for the MDLG hob can be summarized as Figure 7.

## 3. Flow Calculation of MDLG Pump

### 3.1. Derivation of Flow Calculation Formula

During operation, the periodic motion of the internal gear rotor causes the volume of each control chamber within the pump cavity to undergo cyclical changes. Consequently, the instantaneous output flow rate of the gear pump also exhibits periodic pulsation. As a critical output characteristic, the instantaneous flow rate directly correlates with the outlet pressure pulsation of the gear pump. It significantly influences operational properties such as noise, vibration, and reliability of both the pump and the entire hydraulic system. Therefore, this section employs the swept-area method to analyze the instantaneous flow rate of MDLG pumps, providing guidance for selecting partial tooth profile parameters for MDLG design.

**Figure 8:**
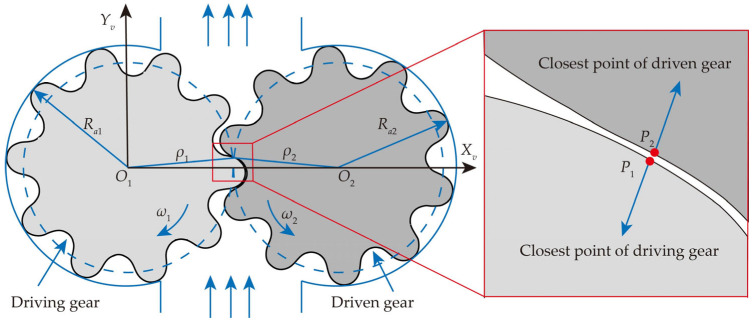
The operating principle of the MDLG pump.

Figure 8 illustrates the operating principle of the MDLG pump. In the figure, *R_a_*_1_ and *R_a_*_2_ represent the tip arc radius of the driving and driven gears, respectively; *ρ*_1_ and *ρ*_2_ are the distances from the meshing point to the centers of the driving and driven gear shafts, respectively.

During an infinitesimal time interval d*t*, the driving gear rotates clockwise by an angle d*φ_v_*_1_, while the driven gear rotates counterclockwise by an angle d*φ_v_*_2_. From the geometric relationship, we derive Equation (20).

(20) $$\left\{\begin{array}{c} \omega_{v1}=\frac{d\varphi_{v1}}{dt}\\ \omega_{v2}=\frac{d\varphi_{v2}}{dt} \end{array}\right.$$

where *ω_vi_* is the angular velocity (rad/s) of gear *i*, and *i* = 1 or 2. Since the gear pair has a transmission ratio of 1, it follows that *φ_v_*_1_ = *φ_v_*_2_.

As illustrated in Figure 8, when the gear of the MDLG pump rotates through an angle d*φ_v_*_1_, the volume change in the discharge chamber in a spur gear pump can be expressed by Equations (21)–(23).

(21) $$dV_{1}=B\left(\frac{1}{2}R_{a1}^{2}d\varphi_{v1}-\frac{1}{2}\rho_{1}^{2}d\varphi_{v1}\right)$$

(22) $$dV_{2}=B\left(\frac{1}{2}R_{a2}^{2}d\varphi_{v2}-\frac{1}{2}\rho_{2}^{2}d\varphi_{v2}\right)$$

(23) $$dV=dV_{1}+dV_{2}=\frac{B}{2}d\varphi_{v1}\left(R_{a1}^{2}+R_{a2}^{2}-\rho_{1}^{2}-\rho_{2}^{2}\right)$$

where d*V*_1_ and d*V*_2_ represent the volumes swept by the tooth profile curves forming the boundary of the discharge chamber on the driving and driven gears, respectively.

To obtain the instantaneous flow rate, calculate the reciprocal of the displacement over time, as shown in Equation (24).

(24) $$Q_{1}=\frac{dV}{dt}=\frac{B\omega_{v1}}{2}\left(R_{a1}^{2}+R_{a2}^{2}-\rho_{1}^{2}-\rho_{2}^{2}\right)$$

**Figure 9:**
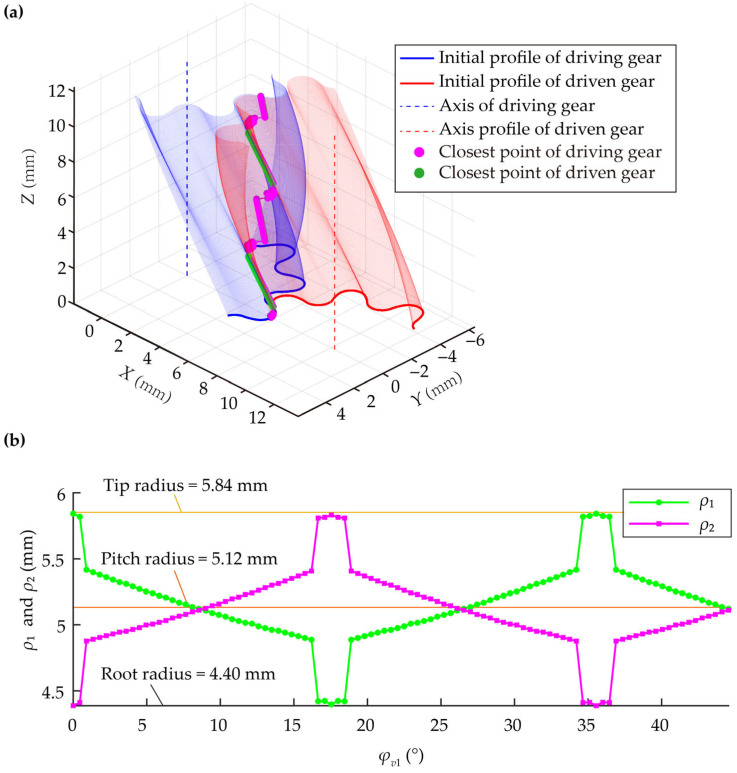
The diagram of the closest points: (**a**) the migration trajectory of the closest point; (**b**) the relationship between *ρ*_1_, *ρ*_2,_ and *φ_v_*_1_.

Since MDLGs are designed based on the spatial curve-meshing principle, not all rotational positions on a cross-section feature have meshing points. Therefore, at each rotational position, the closest points *P*_1_ and *P*_2_ between the profiles of the driving and driven gears are approximated as meshing points. The distances from these closest points to the axis are used as approximate values for *ρ*_1_ and *ρ*_2_. The condition that the closest point needs to satisfy is given by Equation (25). Figure 9a shows the migration trajectory of the closest points *P*_1_ and *P*_2_, while Figure 9b illustrates the relationship between *ρ*_1_, *ρ*_2,_ and *φ_v_*_1_.

(25) $$d_{\min}=\sqrt{{\left(x_{P1}-x_{P2}\right)}^{2}+{\left(y_{P1}-y_{P2}\right)}^{2}}$$

To calculate the instantaneous flow of a helical gear pump, it can be equivalently treated as the integration of an infinite number of infinitely thin spur gear pumps with identical transverse section parameters along the tooth-width direction. Specifically, the MDLG pump is divided into k equal segments along the tooth width, each with a width of *B*/*k*. These segments are then successively superimposed with a certain angular offset along the tooth width. As *k* approaches infinity, this discrete superposition becomes equivalent to integration. Based on the geometry of helical gears, the rotation angle corresponding to the *i*-th infinitely thin spur gear pump is given by Equation (26). Its instantaneous flow rate can be expressed as Equation (27).

(26) $$\varphi_{v}=\theta_{v}-\frac{iB}{k}\frac{\tan\beta }{R}$$

where *θ_v_* is the rotation angle corresponding to the reference transverse section, and *R* is the radius of the gear reference circle.

(27) $$q_{i}=\frac{\omega_{v1}}{2}\left(R_{a1}^{2}+R_{a2}^{2}-\rho_{1i}^{2}-\rho_{2i}^{2}\right)\frac{B}{k}$$

where *ρ*_1*i*_ and *ρ*_2*i*_ are the distances from the closest points to the axis for the driving and driven gears, respectively, in the *i*-th thin gear slice.

Thus, the output instantaneous flow rate of the MDLG pump can be expressed as Equation (28).

(28) $$Q=\sum_{i=0}^{k-1}q_{i}=\sum_{i=0}^{k-1}\frac{\omega_{v1}}{2}\left(R_{a1}^{2}+R_{a2}^{2}-\rho_{1i}^{2}-\rho_{2i}^{2}\right)\frac{B}{k}$$

**Figure 10:**
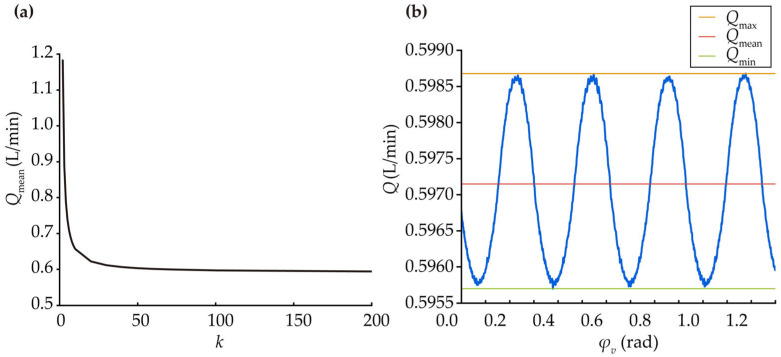
(**a**) Curve of average instantaneous flow rate changing with the number of layers; (**b**) Instantaneous flow change curve at *k* = 100.

Based on the gear parameters set in Section 2.1, a Matlab program was written for the calculations. Figure 10a shows the variation curve of the average instantaneous flow rate with respect to *k* when *ω_v_*_1_ is set to 1000 rpm. The results indicate minimal variation when *k* exceeds 100. Figure 10b displays the output instantaneous flow rate curve of the MDLG pump over four cycles when *k* = 100. It can be observed that the output instantaneous flow rate of the gear pump generally follows a parabolic pattern within a single cycle π/*N*_2_, with an average instantaneous flow rate of 0.5971 L/min.

### 3.2. Simulation Verification

From Equation (23), the outlet area variation rate is given by Equation (29).

(29) $$\frac{dS}{d\varphi_{v}}=\frac{dV}{Bd\varphi_{v}}=\frac{1}{2}\left(R_{a1}^{2}+R_{a2}^{2}-\rho_{1}^{2}-\rho_{2}^{2}\right)$$

**Figure 11:**
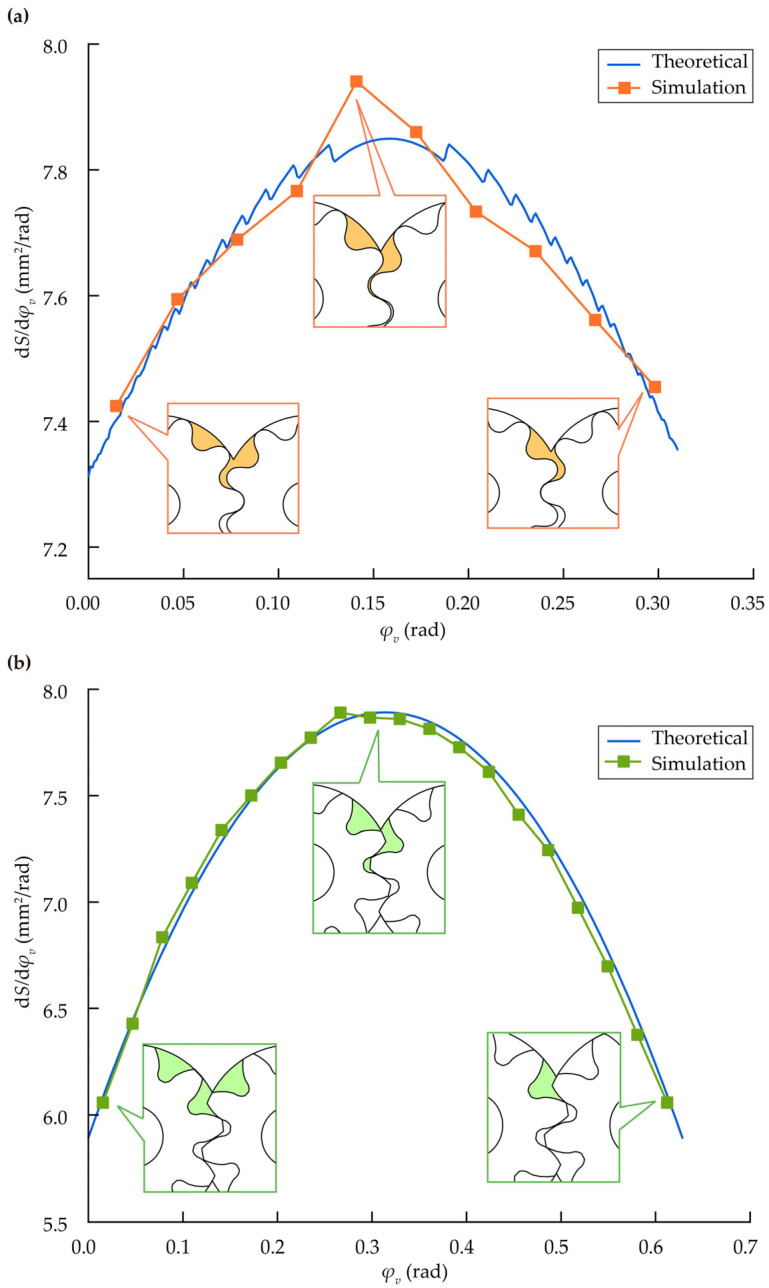
Displacement chamber area change rate: (**a**) MDLG pump; (**b**) involute gear pump.

The MDLG file was imported into SolidWorks 2020 to generate a 3D model of the gear pump. Assemble and extract the gear rotation angle positions at 1.8° intervals. Using the 3D software’s measurement function, the oil discharge chamber area was measured, as shown in Figure 11a. For comparison, a model of an involute gear pump with the same module and tip radius was also simulated under identical conditions, as shown in Figure 11b.

The simulation results indicate that the simulated values of the outlet area variation rate over one cycle align well with the theoretical calculations. Moreover, the results confirm that the MDLG pump exhibits a smaller outlet area variation rate than the comparable involute gear pump.

## 4. Gear Hobbing Simulation and Experiment of MDLG

### 4.1. Virtual Machining Simulation Using Vericut

Following the design and modeling of the hob, a virtual hobbing simulation was conducted to validate its correctness. This section establishes a simulation environment for hobbing operations based on the Vericut platform. Through gear hobbing simulation, the correctness of the hob design is verified.

**Figure 12:**
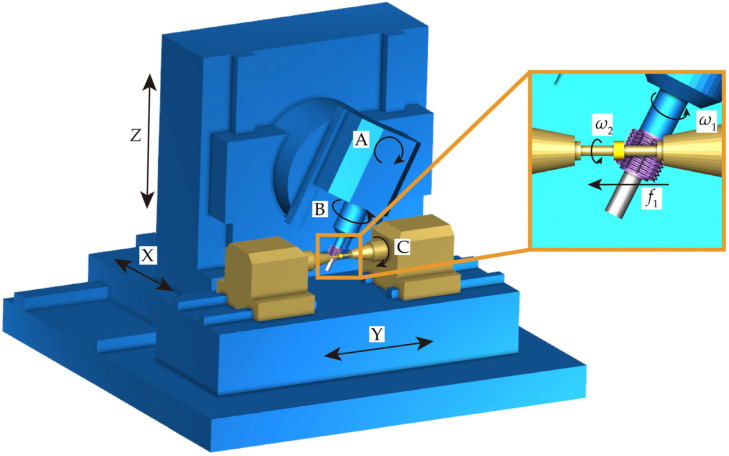
Hobbing machine simulation model.

A simplified 3D model of the gear hobbing machine was constructed. The model incorporates two primary transmission chains. The tool transmission chain consists of the following axes: the B-axis, controlling the hob’s rotation; the A-axis, adjusting the angle between the hob and gear axes; the *X*-axis, controlling the radial cutting depth; and the *Y*-axis, controlling axial feed. The gear drive chain includes the C-axis, which rotates the gear. Figure 12 illustrates the constructed machine-tool simulation model. In the figure, *ω*_1_ (r/min) and *ω*_2_ represent the actual angular velocities of the gear and hob, respectively, while *f*_1_ denotes the feed rate of the hob along the gear axis. Their relationship is defined by Equations (30)–(32).

(30) $$\omega_{2}=\omega_{20}+\omega_{2add}$$

where *ω*_2*add*_ represents the additional motion required for the gear during hobbing, i.e., differential motion.

(31) $$\omega_{20}=\omega_{1}\frac{N_{1}}{N_{2}}$$

where *N*_1_ is the number of threads and *N*_2_ is the tooth number of the gear.

(32) $$\omega_{2add}=f_{1}/n_{2}$$

After setting up the machine tool model, the hob and blank were imported and positioned accordingly. A CNC program was developed based on the motion relationships of each axis. The Siemens sin840d CNC system was used as the machine’s control platform. The simulation parameters were set as follows: tool model tolerance and cutting tolerance were both 0.01 μm, while the interpolation tolerance was 0.005 μm.

**Figure 13:**
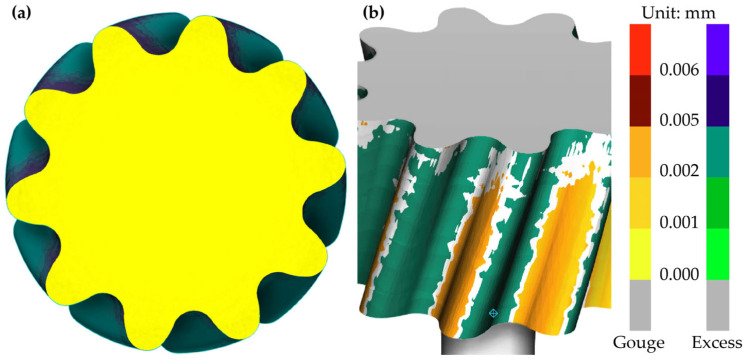
Comparison of MDLG tooth flanks: (**a**) gear after hobbing; (**b**) tooth flank comparison diagram.

First, the hob was rotated counterclockwise by 22.917° around the A-axis to establish a 67.083° angle between the hob and the gear axis. The hob was then adjusted to the appropriate starting position. Next, the hob was fed along the *X*-axis to the predetermined tooth depth, followed by axial feed along the *Y*-axis. During the cutting process, the hob rotated around the B-axis while the gear rotated around the C-axis. The B-axis and C-axis maintained coupled motion. The simulation produced the gear model shown in Figure 13a.

Using Vericut’s automatic comparison function, the theoretical 3D model obtained in Section 2.1 was imported. After setting the comparison tolerance, residual and overcut checks were performed on the tooth flanks, with the results shown in Figure 13b.

The color distribution reveals localized gouge and excess material on the tooth flank, both measuring less than 0.005 mm. These errors primarily arise from the accuracy of 3D modeling and rounding in simulation calculations, falling within acceptable limits. This validates the correctness of the gear hobbing simulation process.

### 4.2. The Hobbing Experiment of MDLGs

**Figure 14:**
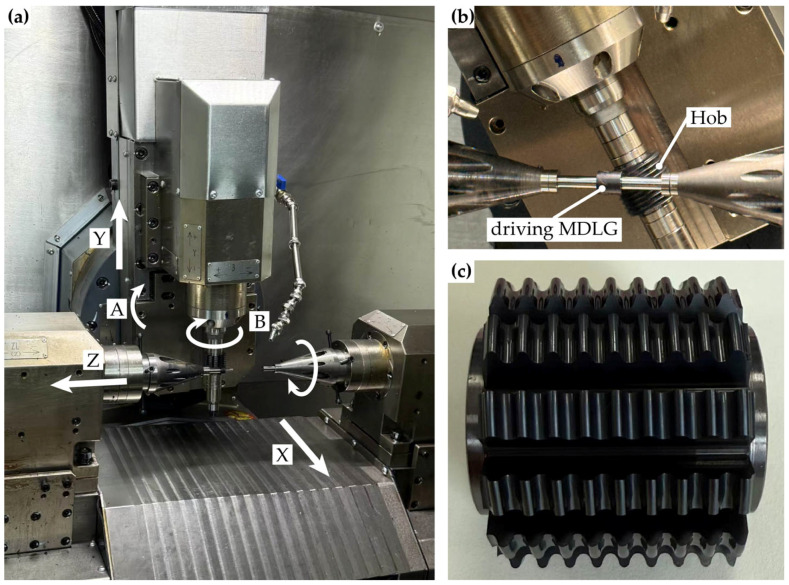
The machine tool and hobbing processes: (**a**) the machine tool; (**b**) the hobbing processes of driving MDLG; (**c**) the manufactured MDLG hob.

Based on the axial profile curve of the hob calculated in Section 2.2.3, an MDLG hob was manufactured, as shown in Figure 14c.

**Figure 15:**
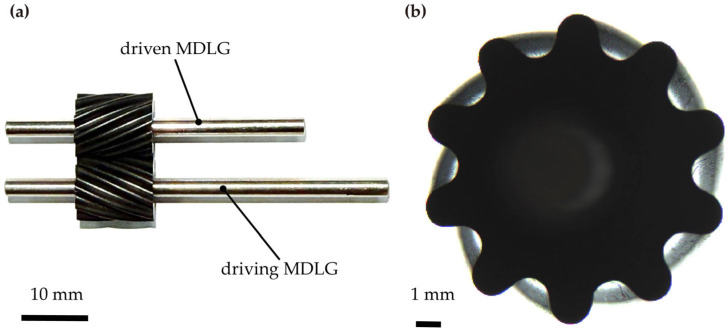
The processed MDLGs: (**a**) the driven MDLG and driving MDLG; (**b**) transverse profile.

A hobbing experiment was conducted on a hobbing machine. During the process, the hob’s rotational speed was set to 900 rpm, with an axial feed rate of 0.2 mm/rev. PEEK was selected as the blank material. The on-site setup of the hobbing experiment is shown in Figure 14a,b. The MDLG produced by the hobbing process is shown in Figure 15.

### 4.3. Accuracy Testing of MDLGs

**Figure 16:**
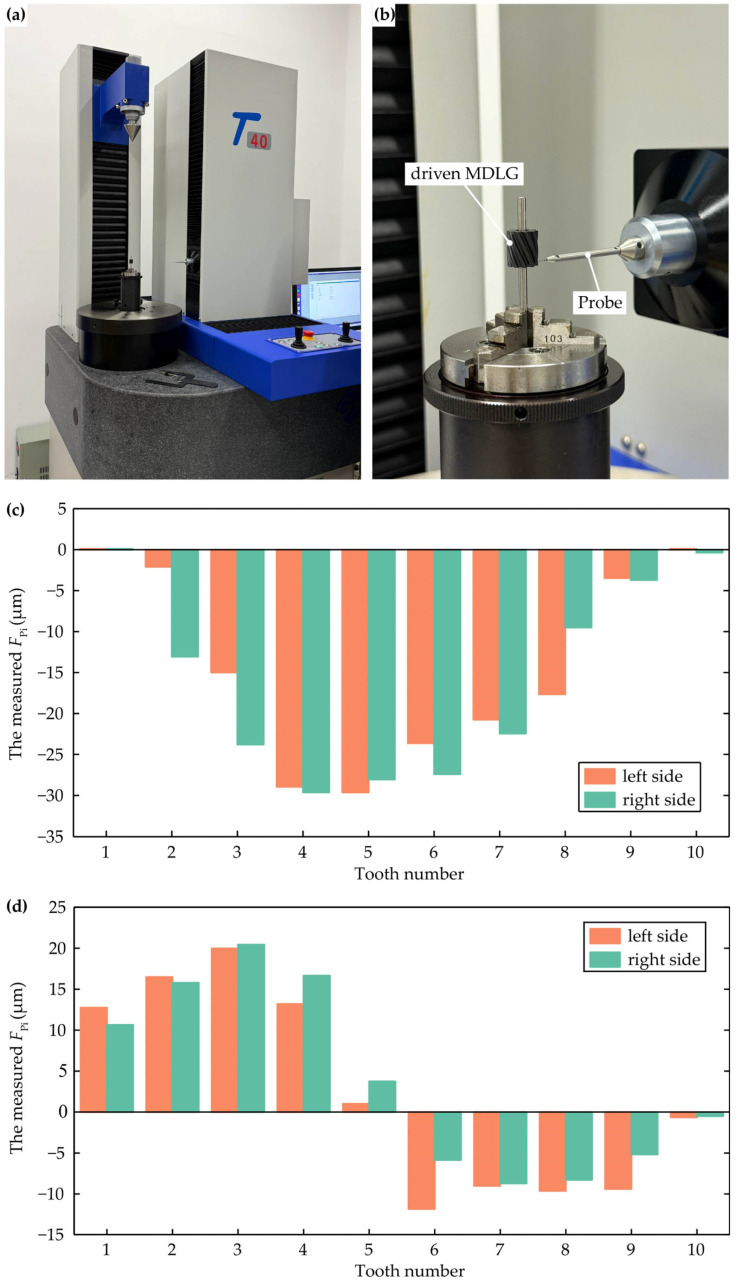
Testing equipment and results: (**a**) testing equipment; (**b**) testing process; (**c**) individual cumulative pitch deviation of driving gear; (**d**) individual cumulative pitch deviation of driven gear.

After deburring and cleaning, the gears underwent precision inspection at the Gear Measurement Center. The measurement focuses on the total cumulative pitch deviations (*F*_p_) of the left and right tooth flanks of the driving and driven gears. Prior to the measurement, gear design parameters and inspection standards must be input into the measurement system. Once clamping is complete, the system automatically collects pitch deviation data. The measurement instrument and results are shown in Figure 16.

According to the international gear standard ISO 1328-1:2013 [29], the total cumulative pitch deviation is defined as the largest algebraic difference between the individual cumulative pitch deviation (*F*_Pi_) values for a specified flank across all teeth of a gear. The *F*_Pi_ values for the driving and driven MDLGs are shown in Figure 16c,d, with the measurement points located on the pitch circle in the middle section along the tooth width direction. The maximum positive and negative deviations of the left side of the driving gear are +0.1 and −29.6 μm, and those of the right side of the driving gear are +0 and −29.6 μm, respectively. The maximum positive and negative deviations of the left side of the driven gear are +20.1 and −12 μm, and those of the right side of the driven gear are +20.3 and −9 μm, respectively. Based on these calculations, the *F*_p_ of the left side of the driving MDLG, the right side of the driving MDLG, the left side of the driven MDLG, and the right side of the driven MDLG are 29.7, 29.6, 32.1, and 29.3 μm, respectively.

Using the formula for total cumulative pitch tolerance as shown in Equation (33), the Grade 7 tolerance for this MDLG is 28.884 μm, while the Grade 8 tolerance is 40.848 μm. The measured total cumulative pitch deviations are all below the Grade 8 tolerance limit, indicating that the machined MDLGs achieve ISO 8 accuracy grade. This validates the feasibility of the proposed hob design and machining process for producing qualified MDLGs. The gear precision deviations primarily stem from hobbing cutter manufacturing deviations, machine tool kinematic accuracy deviations, gear clamping deviations, and plastic deformation during the cutting process.

(33) $$F_{\mathrm{PT}}=\left(0.002d+0.55\sqrt{d}+0.7m_{n}+12\right){\left(\sqrt{2}\right)}^{\left(A-5\right)}$$

where *d* is the pitch circle diameter of the gear, *m*_n_ is the normal module, and *A* is the accuracy class.

## 5. Prototype Design of the MDLG Pump

**Figure 17:**
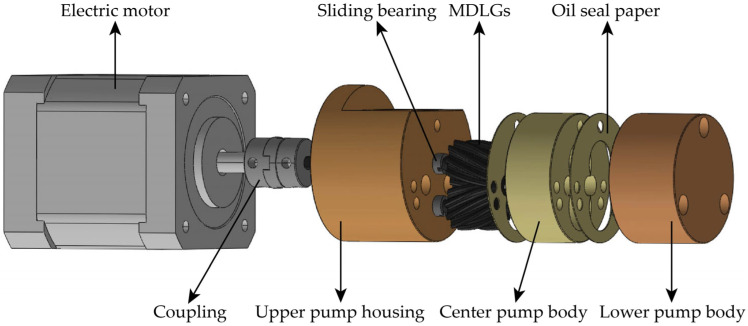
3D structural diagram of the pump.

**Figure 18:**
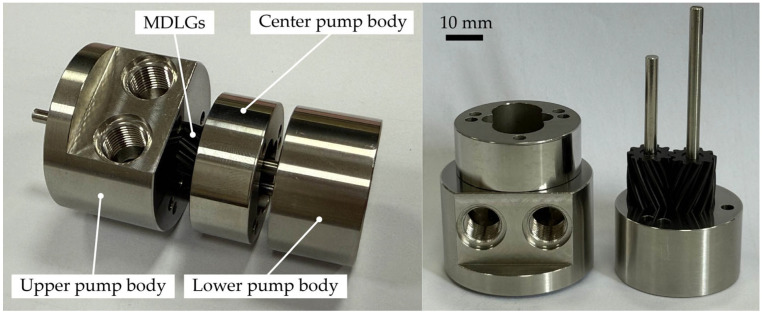
Prototype physical diagram.

The structure of the MDLG pump prototype primarily consists of a pair of MDLGs, an upper pump body, oil-sealing paper, a center pump body, a lower pump body, sliding bearings, an electric rotor, a coupling, and other components. The pump body structure is shown in Figure 17. The remaining pump body sections were machined using conventional machining methods. The overall size of the pump is 38 mm × 38 mm × 55 mm. The physical prototype is depicted in Figure 18.

To facilitate integration with the motor, the pump body adopts a cylindrical structure, with the MDLG positioned at the cylinder’s center. This design allows easy alignment with the motor’s output shaft. Both the driving and driven MDLGs are axially positioned using sliding bearings. The upper and lower pump bodies are aligned with the center pump body using cylindrical pins and secured with connecting bolts. Primary leakage during operation arises from the interfaces between the MDLG end faces and the upper/lower pump body end faces, as well as radial leakage between the gear crown and the pump body bore. Based on conventional gear pump design practice [5,30,31], the radial clearance is set to 0.05 mm, and the end face clearance is controlled within 0.03 mm.

## 6. Conclusions

To address the phenomenon of trapping oil and flow pulsation in the miniature gear pump, a novel miniature double-circular-arc line gear (MDLG) is proposed. And a comprehensive research is conducted on the design and hobbing of MDLGs, theoretical calculation of flow rate, machining, and measuring experiments. The conclusions are as follows:

1. A novel tooth profile of MDLG is proposed on the basis of spatial curve meshing theory, improving the phenomenon of trapping oil in conventional gear pumps.
2. The mathematical model for MDLG hobbing is derived, and the solving algorithm for the hob is developed. And the correctness of the hob is verified by virtual machining simulation.
3. The instantaneous flow rate of the MDLG pump is calculated by using the swept-area method, showing periodic pulsations in the output flow rate.
4. A series of hobbing experiments is conducted on the machine tool. The total cumulative pitch deviation of the manufactured MDLG achieves the accuracy grade of ISO 8.

The design and manufacturing method proposed in this study provide a complete technical solution for the precision manufacturing of MDLG pumps. In the next work, we will focus on the performance testing, CFD optimization of the internal flow field, and reliability studies under high-speed and high-pressure conditions.

## Figures and Tables

**Table 1:** Design parameters for MDLG.

Terms	*m* [mm]	*n* [mm]	*N*_2_	*ζ*	*ρ* [mm]
Value	5.12	11.15	10	1.75	2.5

**Table 2:** Primary design parameters of the hob.

Terms	Value	Units
reference circle radius r1	15.26	mm
number of threads *N*_1_	1	-
pitch of screws *p*_1_	2.925	mm
helix angle *β*_1_	88.222	°
number of flutes	12	-
lead angle	dextral	-

## Data Availability

The original contributions presented in this study are included in the article. Further inquiries can be directed to the corresponding author.
